# Editorial: Advances in neural reprogramming, disease modeling and therapeutic insights

**DOI:** 10.3389/fnagi.2023.1259765

**Published:** 2023-07-28

**Authors:** Shong Lau, Shani Stern

**Affiliations:** ^1^Laboratory of Genetics, Salk Institute for Biological Studies, La Jolla, CA, United States; ^2^Sagol Department of Neurobiology, Faculty of Natural Sciences, University of Haifa, Haifa, Israel

**Keywords:** induced pluripotent stem cells, neurodegeneration, disease modeling, reprogramming, transplants, therapeutics

The human race advanced in the last century in an exponentially growing increase of knowledge. We can send rockets to space, use computers that can easily store terabytes of information and do billions of instructions per second, develop artificial intelligence that is hard to distinguish from a living human person and build smartphones that can communicate dozens of megabytes of information every second between distant places on earth. However, despite major advances in the medical field, many brain-related disorders and diseases still have no cures today, and often we cannot even understand why the disease has started. Disease modeling should therefore be a top priority. Stem cell models and regeneration using stem cells play an important role and hold great promise both for understanding disease and also for cures, especially in neurodegenerative disease. In this Research Topic of Frontiers in Aging Neuroscience, important advances in the field have been reported. This Research Topic includes models for neurodegenerative diseases, potential new therapeutics, and related reviews summarizing what is currently known in the field.

Patient-derived neurons are excellent models but in addition, are now considered also as a huge potential for transplantation and cell replacement in the cases of neurodegeneration or trauma. Limone et al. reviewed the literature on this important topic. The authors cover advances in methods used to differentiate human pluripotent stem cells into several highly specialized types of neurons, including cholinergic, dopaminergic, and motor neurons, and the potential clinical applications of stem cell-derived neurons for common neurodegenerative diseases, including Alzheimer's disease, Parkinson's disease, Huntington's disease, ataxia, and amyotrophic lateral sclerosis. Differentiation techniques for glial cell populations are also described in their review, including oligodendrocytes and microglia. Clinical trials of cell replacement therapies in the nervous system are underway holding great promise in regenerative medicine.

Mesenchymal stem cells have also been shown to incur regenerative possibilities (Guo et al., 2019; Margiana et al., 2022). Nam et al. have sought to explore the regenerative potential of an autologous bone marrow-derived mesenchymal stem cell (BM-MSC) product (Neuronata-R^®^ lenzumestrocel) in amyotrophic lateral sclerosis (ALS). The survival rate of 157 patients treated with intrathecal lenzumestrocel was compared with placebo-treated patients. Remarkedly, the treated patients' survival after 500 and 1,000 days were orders of magnitude higher than the placebo groups, even with a single dose. Therefore, lenzumestrocel treatment is concluded to have a significant, long-term survival benefit in ALS patients.

Neurons derived through the reprogramming of somatic cells and differentiation have been shown to lack aging related signatures and are considered rejuvenated (Mertens et al., 2018). Studies show that even these young neurons carry information that relates to biological differences in the patients' brains (Choudhary et al., 2022; Hussein et al., 2023) and the case of neurodegenerative disease these probably occur long before the disease onset (Stern et al., 2022; Rike and Stern, 2023). However, other studies show also that especially in aging-related diseases, it may be important to use models where the age signatures persist through the differentiation (Mertens et al., 2021; Lau et al., 2022). Aversano et al. reviewed the latest literature on iPSC-derived neurons vs. directly converted neurons from somatic cells that retain the aging information and epigenetic modifications and listed the advantages and disadvantages of these methods.

Autophagy, a cellular process crucial for maintaining cellular homeostasis, takes center stage in the mini-review from Danics et al.. It explores the role of autophagy in aging and age-related neurodegenerative diseases, such as Alzheimer's disease, Parkinson's disease, and Huntington's disease. Dysfunctional autophagy is commonly observed in these diseases, leading to the accumulation of toxic proteins and cellular imbalance. Danics et al. emphasize the potential of targeting autophagy as a therapeutic approach, opening doors to novel pharmacological interventions and clinical trials. Human-relevant models, including induced pluripotent stem cell-derived neurons and directly reprogrammed neurons, offer valuable insights into autophagy alterations and can aid in the development of effective treatments.

Chen et al. highlighted the detrimental effects of a long-term high-fat diet (HFD) on the brain and its link to the activation of specific signaling pathways. The study provides compelling evidence of the molecular pathology underlying obesity-related brain damage and cognitive impairment. It highlights the importance of understanding the impact of HFD on brain metabolism, especially in the context of aging and neurodegenerative diseases. These findings contribute to the development of diagnostic strategies and personalized treatment approaches for age-related conditions and neurodegenerative disorders.

Sleep disturbances have been previously associated with an increased probability of cognitive decline and neurodegeneration (Raggi and Ferri, 2010; Trotti and Karroum, 2016; Wennberg et al., 2017). Wang et al. have investigated Obstructive Sleep Apnea (OSA) that leads to hypoxia and sleep disturbance. The authors used structural equation modeling to investigate how hypoxia and sleep disturbance affect gray matter structures. For this, 74 participants underwent overnight polysomnography and T1-weighted Magnetic Resonance Imaging. The computational models revealed hypoxia-associated increases in gray matter volume, cortical thickness, and sulcal depth. Sleep disturbance on the other hand was shown to be largely associated with reduced gray matter volume and sulcal depth.

The dysfunction of BDNF signaling has central roles in neurological disorders including psychiatric and neurodegenerative diseases (Boulle et al., 2012; Lima Giacobbo et al., 2019; Miranda et al., 2019). Li Y. et al. explored the impact of Brain-derived neurotrophic factor (BDNF) dysfunctions that are mediated by the activation of two receptors, tropomyosin receptor kinase B (TrkB) receptor and the p75 neurotrophin receptor and are involved in physiological and pathological processes throughout life. Their review discusses the current knowledge and future directions in BDNF-associated research with an emphasis on the physiological and pathological functions in a long list of neurodegenerative diseases as well as other diseases such as diabetes and cancer. Methods to increase BDNF levels are discussed and BDNF-overexpressed stem cell transplantation is suggested as a possible promising therapeutic strategy.

In the study of Kühne et al. an *in vitro* rabbit neurosphere model was introduced to study the effects of intrauterine growth restriction (IUGR) on neuronal development. IUGR is known to cause neurodevelopmental abnormalities, and this model mimics the conditions seen in humans. The neuroprotective properties of lactoferrin, a potential therapeutic agent, were highlighted in preventing adverse effects on neurite length associated with IUGR. These findings provide valuable insights into the development of interventions to protect against IUGR-induced alterations in neuronal development and improve long-term cognitive outcomes.

Lin et al. unravel the role of C-X-C motif chemokine 12 (CXCL12) in experimental autoimmune encephalomyelitis (EAE), an animal model of multiple sclerosis (MS). The team used gene therapy to upregulate CXCL12 in the spinal cord of rats and induced EAE. They found that upregulated CXCL12 alleviated EAE symptoms and reduced clinical scores. The levels of myelin basic protein (MBP) and glial fibrillary acidic protein (GFAP) were higher in the CXCL12 group, indicating enhanced remyelination. Interestingly, the upregulation of CXCL12 did not induce significant leukocyte infiltration into the spinal cord. The study also demonstrated that CXCL12 promoted the differentiation of OPCs into oligodendrocytes *in vitro*. This study offers new avenues for targeting the CXCL12/CXCR4 axis as a potential therapeutic approach to enhance remyelination in MS and sheds light on the underlying mechanisms involved.

Contrast-induced encephalopathy (CIE) is a rare complication arising from exposure to iodinated contrast media during angiographic procedures. The review from Zhang et al. provides insights into the risk factors associated with prognosis. Female gender, younger age, higher contrast medium dose, and cerebral angiography procedure were linked to a poorer prognosis. Comorbidities such as hypertension, diabetes mellitus, renal insufficiency, and previous reactions to contrast media were also identified as potential risk factors. Neuroimaging and cerebrospinal fluid examination can aid in diagnosing CIE and distinguishing it from other neurological conditions. Supportive care, hydration, steroids, mannitol, and anti-epileptic medications are common treatment approaches. The review emphasizes that CIE can have lasting neurological effects, even with low doses of contrast media, and highlights the need for further research to better understand and manage this condition.

Meige's syndrome is a rare form of cranial dystonia. Current treatments such as oral medications and botulinum toxin injections have limitations. Deep brain stimulation is an alternative but has technical requirements and high costs. Huang et al. explore the use of CT-guided extracranial radiofrequency ablation for Meige's syndrome. They performed radiofrequency ablation on cranial nerves involved in the condition, targeting specific muscles. They found that the treatment resulted in symptom alleviation, although some patients experienced mild facial paralysis and sensory changes. Recurrences were observed in two cases. They suggested that CT-guided extracranial radiofrequency ablation could be a potential treatment option for Meige's syndrome, in addition to botulinum toxin injections and deep brain stimulation.

A major complication of liver disease is Hepatic encephalopathy. Through elevated levels of ammonia/ammonium in the blood and cerebrospinal fluid, the patients suffer from olfactory dysfunction. Li M. et al. performed patch-clamp recordings of mitral cells (MCs) in the mouse olfactory bulb (OB) and found involvement of glutamate receptors in NH${}_{4}^{+}$-induced hyperexcitability of MCs. NH${}_{4}^{+}$ reduced the currents of voltage-gated K^+^ channel (Kv) that may be linked to an attenuation of the spontaneous firing amplitudes. NH${}_{4}^{+}$ also enhanced the amplitude of long-lasting spontaneous excitatory post-synaptic currents (sEPSCs) and increased the expression of NR1 and GluR1 on the membrane indicating an increased trafficking of glutamate receptors to the membrane. Importantly, the enhanced activity of glutamate receptors caused increased cell death through excitotoxicity, providing a potential pathological mechanism of the olfactory defects in patients with hyperammonemia and HE.

## Author contributions

SS: Conceptualization, Writing—original draft, Writing—review and editing. SL: Conceptualization, Writing—original draft, Writing—review and editing.
